# Bacterial Diversity in Sediments from Lianhuan Lake, Northeast China

**DOI:** 10.3390/microorganisms12091914

**Published:** 2024-09-20

**Authors:** Wenmiao Pu, Mingyu Wang, Dan Song, Wei Zhao, Xuran Sheng, Tangbin Huo, Xue Du, Xin Sui

**Affiliations:** 1 Engineering Research Center of Agricultural Microbiology Technology, Ministry of Education & Heilongjiang Provincial Key Laboratory of Ecological Restoration and Resource Utilization for Cold Region & Key Laboratory of Microbiology, College of Heilongjiang Province & School of Life Science, Heilongjiang University, Harbin 150080, China; puwenmiao@163.com (W.P.); wmy022234@163.com (M.W.); 2 Heilongjiang River Basin Fishery Ecological Environment Monitoring Center, Ministry of Agriculture and Rural Affairs, Heilongjiang River Fisheries Research Institute, Chinese Academy of Fishery Sciences, Harbin 150010, China; sodafish2024@163.com (D.S.); zw19802568331@163.com (W.Z.); sxr3584802@163.com (X.S.); tbhuo@163.com (T.H.); 3 College of Marine Science and Environment, Dalian Ocean University, No. 52, Heishijiao Street, Shahekou District, Dalian 116023, China; 4 College of Animal Science and Technology, Sichuan Agricultural University, Chengdu 611130, China

**Keywords:** Lianhuan Lake, sedimental bacteria, physicochemical property

## Abstract

Lake microbiota play a crucial role in geochemical cycles, influencing both energy flow and material production. However, the distribution patterns of bacterial communities in lake sediments remain largely unclear. In this study, we used 16S rRNA high-throughput sequencing technology to investigate the bacterial structure and diversity in sediments across different locations (six independent lakes) within Lianhuan Lake and analyzed their relationship with environmental factors. Our findings revealed that both the alpha and beta diversity of sediment bacterial communities varied significantly among the six independent lakes. Furthermore, changes between lakes had a significant impact on the relative abundance of bacterial phyla, such as Pseudomonadota and Chloroflexota. The relative abundance of Pseudomonadota was highest in Habuta Lake and lowest in Xihulu Lake, while Chloroflexota abundance was lowest in Habuta Lake and highest in Tiehala Lake. At the genus level, the relative abundance of *Luteitalea* was highest in Xihulu Lake compared to the other five lakes, whereas the relative abundances of *Clostridium*, *Thiobacillus*, and *Ilumatobacter* were highest in Habuta Lake. Mantel tests and heatmaps revealed that the relative abundance of Pseudomonadota was significantly negatively correlated with pH, while the abundance of Chloroflexota was significantly positively correlated with total phosphorus and total nitrogen in water, and negatively correlated with electrical conductivity. In conclusion, this study significantly enhances our understanding of bacterial communities in the different lakes within the Lianhuan Lake watershed.

## 1. Introduction

Freshwater lake ecosystems play a pivotal role in the global biogeochemical cycle [1,2]. Various interconnected factors, including physical, chemical, and biological components, influence the structure and function of lake ecosystems [3,4]. For instance, higher biodiversity within a freshwater lake ecosystem can directly contribute to its stability through resource use complementarity and functional redundancy [5,6]. Thus, diversity, including species richness and evenness, helps preserve the functional stability of biological communities [7,8,9]. However, many freshwater lakes have undergone significant human-induced environmental changes, such as urbanization, deforestation, and habitat fragmentation [10,11], which have led to substantial disturbances [12,13,14]. Therefore, understanding and preserving the biological components of freshwater lake ecosystems is essential for maintaining their structure, function, and long-term sustainability. Sediment is an important component of freshwater lake ecosystems, originating from the weathering and erosion of organic matter, soils, and other fluvial sources. Sediment composition is influenced by bedrock and surrounding soils, which are eroded into water bodies through processes such as wind and rainfall. Sediments significantly impact aquatic and riparian ecosystems, as many organisms rely on them for survival and reproduction [15,16].

Bacteria are widely distributed in sedimentary environments and play a central role in the food web, forming distinct trophic levels and inducing cascading effects [17]. Previous studies have shown that sediment bacterial communities and their composition may vary spatially in response to factors such as hydrological variation, climate change, water quality, land use, and habitat heterogeneity [3,18]. Despite these insights, there remains a significant gap in our understanding of how these factors interact to influence sedimentary bacterial communities within multi-lake systems. These systems, which often consist of multiple interconnected lakes, present unique and complex sedimentary environments. While large-scale spatial patterns of bacterial communities have been extensively studied in multi-lake systems, fine-scale variations and the temporal dynamics of bacterial communities remain largely underexplored [19,20]. This lack of understanding hinders our ability to predict ecological responses to environmental changes and develop effective lake management and conservation strategies.

Lianhuan Lake (124°0′–124°14′ E, 46°32′–46°49′ N), located in Heilongjiang Province, China, is the largest brackish lake in the region, consisting of 18 independent lakes with distinct characteristics [21]. However, increasing surface water connectivity due to human activities has accelerated eutrophication in these smaller lakes [22]. In response, many studies have focused on phytoplankton, zooplankton, and aquatic macroinvertebrates in connected lake ecosystems [21,23,24]. Dou et al. (2022) reported that macroinvertebrate community structure and diversity changed significantly across different spatial and temporal scales in Lianhuan Lake and identified pH and total phosphorus (TP) as key environmental factors driving these changes [21]. Dou et al. (2024) also reported that the secondary productivity of macroinvertebrates exhibited significant spatiotemporal variation in Lianhuan Lake, with pH, TP, conductivity, and chlorophyll a (Chla) identified as key environmental factors [25]. However, the spatiotemporal variations in sediment microbial communities and diversity in Lianhuan Lake remain unclear. Additionally, it is unknown whether the distribution patterns of sediment microbial communities align with the findings of previous research [21,25].

In this study, we selected six independent lakes within the Lianhuan Lake watershed to investigate the composition of sediment bacterial communities using Illumina high-throughput sequencing technology. Additionally, we analyzed the relationships between bacterial communities and the physicochemical properties of the water and sediment. Our objectives were to (1) identify the difference in sediment bacterial communities across these six independent lakes and (2) clarify the environmental factors driving sediment bacterial communities in each lake. The results of this study are crucial for understanding water quality in the context of global climate change and for providing scientific data to explore changes in water-bacteria communities in Lianhuan Lake. This research also lays the foundation for future studies on nutrient cycling.

## 2. Materials and Methods

### 2.1. Study Area

This study focuses on six small lakes located in the low-lying center of the Songnen Plain in northeastern China. These lakes have an average depth of 2.14 m and a maximum depth of 4.6 m, with a surface area of 580 km^2^. Rapid economic and technological development has significantly increased agricultural activities around the Lianhuan Lakes. Consequently, pollutants such as fertilizers and animal waste from these activities are discharged directly into the lakes via surface runoff, without undergoing any treatment. This study evaluated six lakes within the Lianhuan Lake system: Habuta, Delongpao, Xihulu, Nashidai, Huoshaohei, and Tiehala. These lakes represent major water bodies in the upper, middle, and lower reaches of Lianhuan Lake and were selected to assess the effects of environmental variables on microorganisms. The lakes have different natural morphologies and exhibit distinct characteristics. They are interconnected and vary in their geographic locations within Lianhuan Lake.

### 2.2. Sediment and Water Sampling

**Figure 1:**
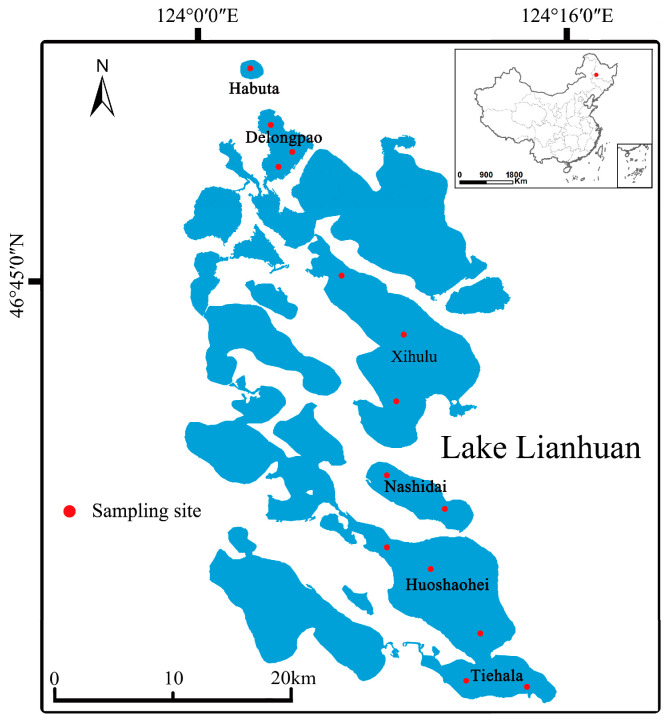
Geographical locations of the lakes examined in this study.

Surface sediment samples were collected in August 2020 from the six lakes of Lianhuan Lake (Figure 1). The number of samples per lake ranged from one to three, depending on environmental heterogeneity and lake size. At each sampling site, surface sediments were collected using a pre-cleaned grab sampler from an area of 0.25 m × 0.35 m to a depth of 0.1 m. Three sediment quadrats were selected at each site. In each quadrat, five sub-samples of sediment, each weighing approximately 100 g, were collected and combined into a single composite sample (Figure S1) [26]. These composite samples were immediately placed into sterile plastic bags on dry ice and transported to the laboratory. Upon arrival, each composite sample was divided into two portions. One portion was placed in 15 mL sterilized polypropylene tubes and stored at −80 °C for DNA extraction, while the other portion was air-dried, sieved through a 2 mm mesh, and used for chemical analysis [27].

For water samples, sampling began by rinsing a polyethylene plastic bucket three times with in situ water. After rinsing, the bucket was used to mix three water samples, from which a 1.5 L composite water sample was drawn. This composite sample was then filtered through a 200 μm nylon filter to remove insoluble impurities and collected in the polyethylene plastic bucket. Immediately after collecting the 1.5 L of water at each site, the samples were transported to the laboratory at a low temperature (0–4 °C). Once in the laboratory, the samples were used for physicochemical analysis [28].

Water temperature (WT), dissolved oxygen (DO), pH, and conductivity were measured in the field using a YSI multiparameter water quality detector (YSI Incorporated, Yellow Springs, OH, USA). The remaining parameters were measured in the laboratory using standard procedures. Specifically, total phosphorus in water (WTP) was determined using the ammonium molybdate spectrophotometric method [29]. Total nitrogen in water (WTN) was measured by UV spectrophotometry following alkaline potassium persulfate digestion [29]. Ammonia (NH_4_^+^-N) was measured using Nessler’s reagent (BKMAM-Lab Ltd., Changde, China) spectrophotometry (T6 New Century, Beijing Persee General Instrument Co., Ltd., Beijing, China) [30], and nitrate (NO_3_^−^-N) [31], nitrite (NO_2_^−^-N) [32], and Chla [33] were all measured using spectrophotometric methods (T6 New Century, Beijing Persee General Instrument Co., Ltd., China). Total suspended solids (TSSs) were assessed using the gravimetric method [34], and chemical oxygen demand (COD) was measured using the permanganate (CODMn) method [35]. Additionally, sediment total carbon (TC) and total nitrogen (TN) were measured using an elemental analyzer (Vario EL III, Elementar, Langenselbold, Germany) [36], and the TP content in the sediments was determined using perchloric acid digestion followed by analysis with a UV-2550 spectrophotometer (Shimadzu, Kyoto, Japan).

### 2.3. DNA Extraction, PCR Amplification, and MiSeq Sequencing

Genomic DNA from all samples was extracted using the FastDNA Spin Kit (MP Biomedicals, Santa Ana, CA, USA) according to the manufacturer’s instructions [37]. The V3-V4 hypervariable region of the bacterial 16S rRNA gene was amplified using universal bacterial primers 341F (5′-CCT AYG GGR BGC ASC AG-3′) and 806R (5′-GGA CTA CHV GGG TWT CTA AT-3′) (Sangon Biotech Co., Ltd., Shanghai, China), with a sample-specific 12 bp barcode added to 341F [38]. PCR amplification was performed using TransGen AP221-02 TransStart FastPfu DNA polymerase in a 20 μL reaction system (TransGen Biotech Co., Ltd., Beijing, China). The PCR protocol included an initial denaturation at 95 °C for 2 min, followed by 35 cycles of denaturation at 95 °C for 10 s, annealing at 60 °C for 30 s, extension at 72 °C for 30 s, and a final extension at 72 °C for 5 min.

PCR products were detected via 2% agarose gel electrophoresis and recovered using the AMPure XT Beads DNA Gel Extraction Kit (Beckman Coulter Genomics, Danvers, MA, USA), according to the manufacturer’s instructions. The products were quantified using a Quantus^TM^ Fluorometer (Promega, Madison, WI, USA). The purified PCR amplicons were pooled in equimolar amounts and paired-end sequenced by Majorbio Bio-Pharm Technology Co. Ltd. (Shanghai, China) on an Illumina MiSeq PE300 sequencer (Illumina, San Diego, CA, USA) following standard protocols [39]. The sequences were submitted to the NCBI database under accession number PRJNA1140916.

### 2.4. Bioinformatics and Statistical Analyses

The data were denoised with DADA2 using the ‘qiime dada2 denoise-paired’ command to obtain amplicon sequence variants (ASVs) [40]. Afterward, the ASV feature sequences and ASV tables were merged, and singleton ASVs were removed. Alpha and beta diversity analyses were performed based on the obtained ASV feature sequences and abundance tables. The SILVA database [41] was used to annotate species from the NT-16S database based on the ASV feature sequences, and the abundance of each species in each sample was determined using the ASV abundance table. The confidence threshold for annotation was set at 0.7.

Differences in sediment and water physicochemical properties among the six lakes were identified with SPSS (version 26) using one-way analysis of variance (ANOVA) coupled with Duncan’s test. Alpha diversity indices, including the ACE index, Chao1 index, Shannon index, and Simpson index, were calculated using the ‘vegan’ package (version 2.6.6.1) in R (version 4.4.1). The ‘vegan’ package was also used for non-metric multidimensional scaling (NMDS) analyses of microbial beta diversity based on Bray–Curtis dissimilarity. Permutational multivariate analysis of variance (PERMANOVA) was conducted to test differences in bacterial community structure among lakes, and the Kruskal–Wallis test was used to analyze differences in species abundance among multiple groups [42]. Linear discriminant analysis (LDA) effect size (LEfSe) was performed using the ‘microeco’ package (version 1.8.0) with a threshold of 3.8, as described by Yu et al. (2023), to identify bacterial families with statistically significant differences in sediment across the lakes [43]. Redundancy analysis (RDA) was conducted using the Euclidean distance metric in the ‘microeco’ package [44]. Additionally, the Mantel test was performed using the ‘linkET’ package (version 0.0.7.4) in R to assess the correlation between bacterial community diversity, richness, and physicochemical properties of both water and sediment [45]. The Mantel test was conducted using the ‘microeco’ package (version 1.8.0) in R software (version 4.4.1) to assess whether the relationship between the soil microbial community composition and α-diversity is correlated with soil physicochemical properties. We selected the sediment microbial Shannon index and richness index to represent “Diversity” and “Richness”, respectively, and Microbial Bray–Curtis dissimilarity to represent “Composition”.

All data in this study were analyzed using SPSS 26.0 (SPSS Inc., Chicago, IL, USA) and R 4.4.1 (Teem 2024). Graphs were generated and analyzed using GraphPad Prism 7.0 (GraphPad Software, Inc., San Diego, CA, USA) and R 4.4.1 (Teem 2024). A *p*-value < 0.05 was considered statistically significant.

## 3. Results

### 3.1. Water and Sedimental Physicochemical Characteristics

The physicochemical properties of the water (WT, DO, pH, conductivity, NH_4_^+^-N, WTP, WTN, NO_2_^−^-N, NO_3_^−^-N, Chla, and SS) varied significantly among the different lakes (Table 1, *p* < 0.05), except for the content of COD, which did not show significant differences (Table 1, *p* > 0.05). WT and DO levels in Xihulu Lake and Habuta Lake were significantly lower than in the other four lakes, while Habuta Lake exhibited the lowest pH among all the lakes. Conductivity in Delongpao Lake was the lowest compared to all other lakes, and its NH_4_^+^-N content was significantly lower than in Habuta Lake, Xihulu Lake, Nashidai Lake, and Tiehala Lake. WTP in Nashidai Lake was significantly higher than in the other five lakes, while WTN levels were highest in Delongpao Lake. The NO_2_^−^-N concentration in Habuta Lake was higher than in Delongpao, Xihulu, Huoshaohei, and Tiehala lakes, and NO_3_^−^-N levels were also higher in Habuta Lake compared to the other lakes. Chla concentrations in Huoshaohei Lake were the lowest, while SS levels were significantly higher than in the Habuta and Delongpao lakes (Table 1, *p* < 0.05).

Similarly, the sediment physicochemical properties (TN, TC, TP) also showed significant differences across the six independent lakes (Table 2, *p* < 0.05). Habuta Lake exhibited the lowest TC levels, whereas TN and TP concentrations were the highest in Delongpao Lake (Table 2, *p* < 0.05).

### 3.2. Bacterial Diversity in the Sediments of Different Lakes

**Figure 2:**
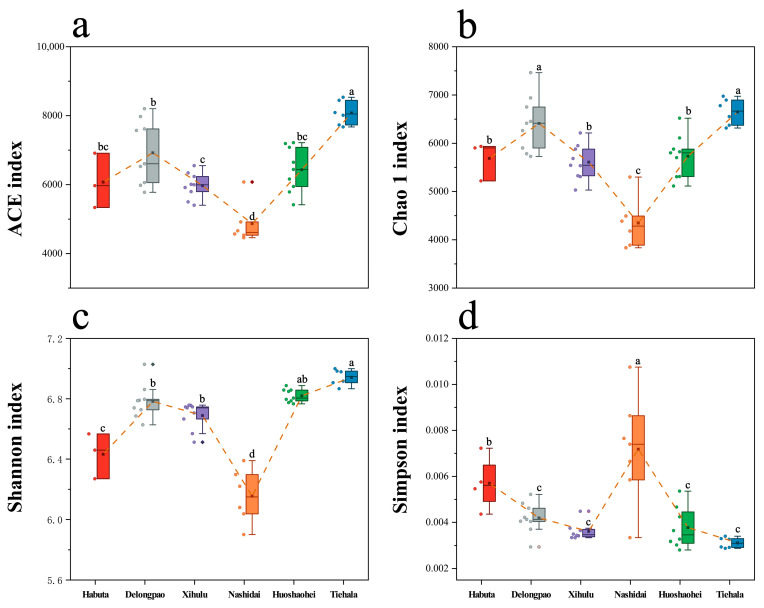
Boxplots with error bars showing the alpha diversity indices of bacterial communities in the sediments of Lianhuan Lake. (**a**) ACE index; (**b**) Chao1 index; (**c**) Shannon index; and (**d**) Simpson index. Lowercase letters indicate significant differences (*p* < 0.05) between sampling groups. Significant variations between experimental groups were identified via ANOVA followed by the Waller–Duncan post hoc test for pairwise comparisons.

The alpha diversity of the sediment bacterial communities varied significantly across the six lakes (Figure 2, *p* < 0.05). Nashidai Lake exhibited the lowest ACE, Chao1, and Shannon indices compared to the other lakes (Figure 2, *p* < 0.05). In contrast, Nashidai Lake had the highest Simpson index among all the lakes. Additionally, there was no significant difference in the ACE and Chao1 indices between Habuta and Huoshaohei Lakes (Figure 2).

### 3.3. Bacterial Community Composition in the Sediments of Different Lakes

**Figure 3:**
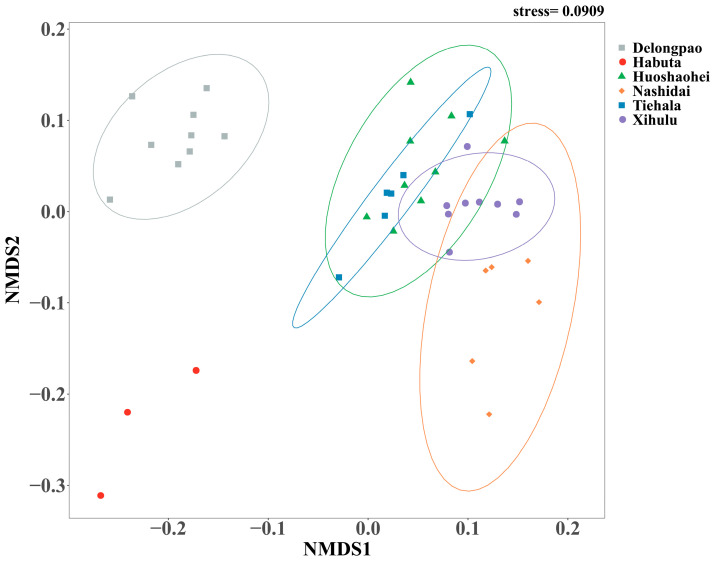
NMDS analysis of microbial beta diversity based on Bray–Curtis distance metrics. Different colored dots represent the bacterial communities of different lakes. Circles indicate the 95% confidence intervals around the bacterial community estimates. The color of the circles represents different lakes. The stress value reflects the error between the original distances and the low-dimensional spatial distances obtained through NMDS.

The bacterial community structures, including both unique and shared ASVs, were analyzed among the sediment samples from the six lakes. A total of 2275 ASVs were shared between all lakes. Delongpao Lake exhibited the highest number of unique ASVs (1246), followed by Xihulu Lake (319), Habuta Lake (281), Huoshaohei Lake (239), Tiehala Lake (154), and Nashidai Lake (145). NMDS analysis based on Bray–Curtis dissimilarity revealed significant differences in the beta diversity of sediment bacterial communities across the six lakes (Figure 3). The PERMANOVA results further confirmed these differences, showing significant variation in bacterial community structures between the lakes (PERMANOVA R^2^ = 0.67, *p* = 0.001), indicating that at least one lake has a markedly distinct bacterial community structure. Pairwise comparisons of the bacterial communities highlighted distinct compositions between specific lakes, with adjusted *p*-values confirming significant separations. However, no significant difference was observed between Huoshaohei Lake and Tiehala Lake (adjusted *p*-value = 0.063) (Table 3).

**Figure 4:**
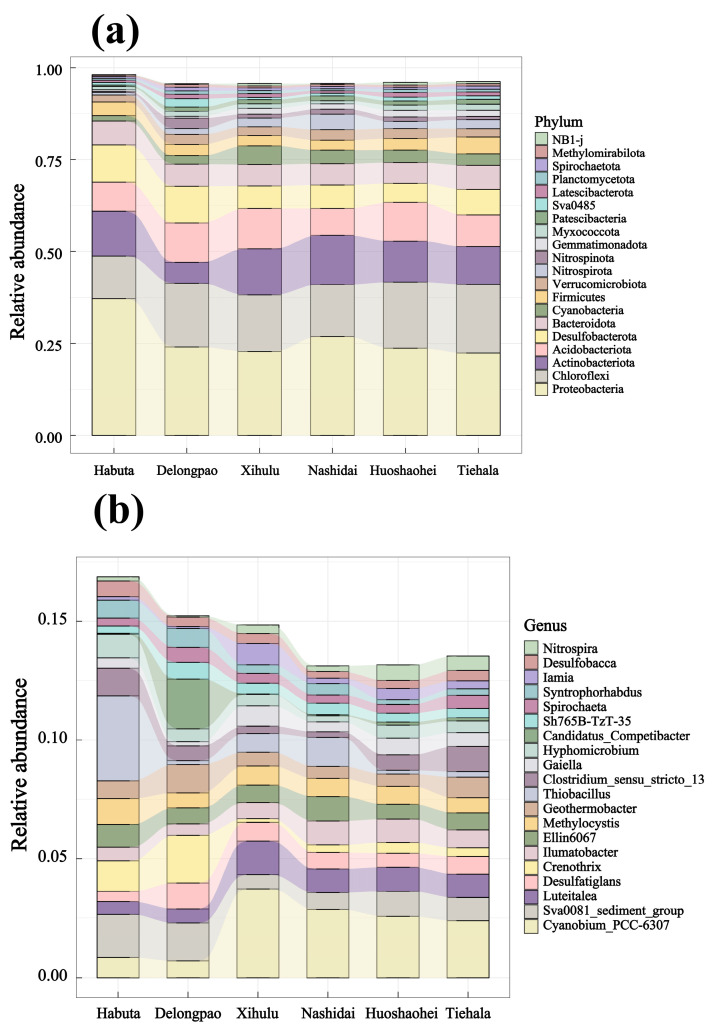
Relative abundance of the top 20 bacterial phyla and genera in different lakes of Lianhuan Lake: (**a**) bacterial phyla; (**b**) bacterial genera.

**Figure 5:**
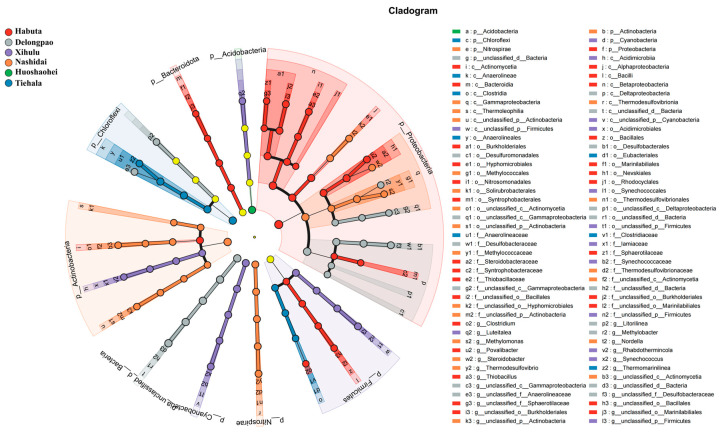
LEfSe analysis of ASVs among the six lakes (Habuta, Delongpao, Xihulu, Nashidai, Huoshaohei, and Tiehala). The circles represent taxonomic levels from Kingdom to Genus, radiating from inside to outside. Nodes in different colors indicate microbial taxa significantly enriched in the corresponding group and contributing to intergroup differences. Yellow nodes represent taxa that are not significantly different among the groups or have no significant effect on intergroup differences.

The relative abundance of both phyla and genera varied significantly among the different lakes (Figure 4, *p* < 0.05). At the phylum level, Pseudomonadota was dominant in all lake sediments. However, at the genus level, *Cyanobium_PCC-6307* and *Thiobacillus* exhibited significant differences across the lakes. LEfSe analyses were also conducted to explore the taxa (from phylum to genus level) affected by the changes in lake environments (Figure 5). The results indicated that a total of 90 bacterial taxa showed significant differences in relative abundance across the six lakes, as evidenced by LDA effect size scores greater than 3.8.

### 3.4. Relationships between Sediment Bacterial Communities and Environmental Factors

**Figure 6:**
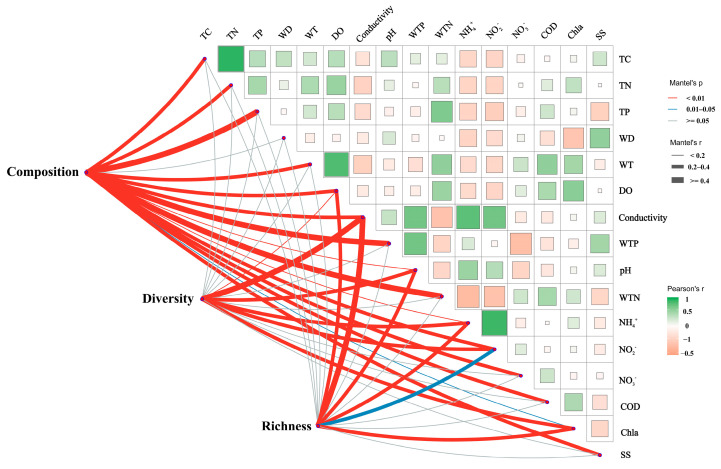
Mantel analysis illustrating the relationships between sediment bacterial community composition and alpha diversity with water and sediment physicochemical properties. The pink and blue lines indicate varying levels of correlation, while the gray lines represent no correlation. The thickness of the lines corresponds to Spearman’s correlation coefficients; thicker lines indicate stronger correlations, whereas thinner lines represent weaker correlations. TC: total carbon; TN: total nitrogen; TP: total phosphorus; WT: water temperature; DO: dissolved oxygen; Conductivity: electrical conductivity; pH: *pondus hydrogenii*; WTP: total phosphorus in water; WTN: total nitrogen in water; NH_4_^+^: ammonia nitrogen; NO_3_^−^: nitrate nitrogen; NO_2_^−^: nitrous nitrogen; COD: chemical oxygen demand; Chla: chlorophyll a; SS: suspended solids; Diversity: sediment microbial Shannon index; Richness: sediment microbial richness; Composition: microbial Bray–Curtis dissimilarity.

Mantel’s test revealed significant correlations between the composition, diversity, and richness of sediment bacterial communities and the physicochemical properties of the water and sediment (Figure 6). Specifically, bacterial community composition, diversity, and richness were significantly correlated with several physicochemical factors, including conductivity, WTP, and NH_4_^+^. Additionally, bacterial composition showed correlations with TC, TN, TP, WT, DO, pH, WTN, NO_2_^−^, NO_3_^−^, COD, and SS. The Shannon index, which measures bacterial diversity, was correlated with Chla. Bacterial richness was found to correlate with NO_3_^−^ and Chla.

**Figure 7:**
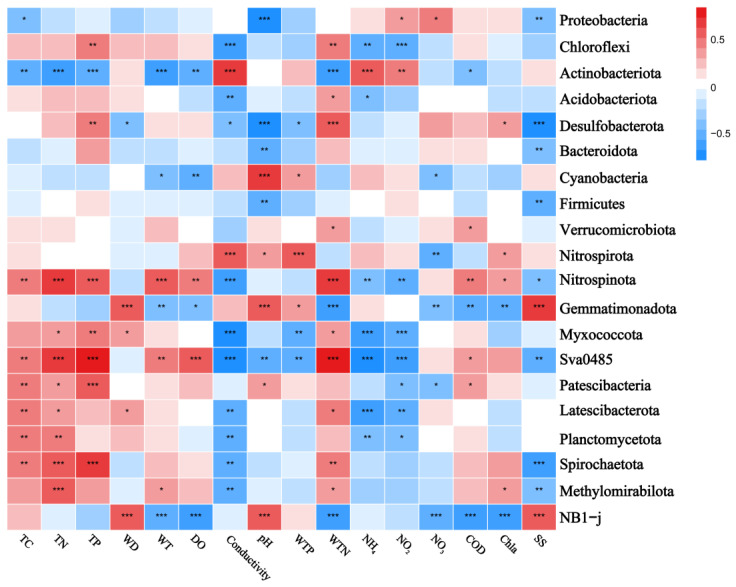
Correlation heatmap of the top 20 ranked bacterial phyla and water and sediment physicochemical properties across different lakes. Positive and negative correlations are indicated in red and blue, respectively (‘*’ indicates 0.01 < *p* ≤ 0.05; ‘**’ indicates 0.001 < *p* ≤ 0.01; ‘***’ indicates *p* ≤ 0.001). Correlations between variables were calculated using the Pearson correlation coefficient. TC: total carbon in sediment; TN: total nitrogen in sediment; TP: total phosphorus in sediment; WT: water temperature; DO: dissolved oxygen; Conductivity: electrical conductivity; pH: pondus hydrogen; WTP: total phosphorus in water; WTN: total nitrogen in water; NH_4_^+^: ammonia nitrogen; NO_3_^−^: nitrate nitrogen; NO_2_^−^: nitrous nitrogen; COD: chemical oxygen demand; Chla: chlorophyll a; SS: suspended solids. The R values are displayed in various colors within the heatmap, with *p*-values below 0.05 marked by an asterisk (*). The legend on the right shows the color scale for different R values.

**Figure 8:**
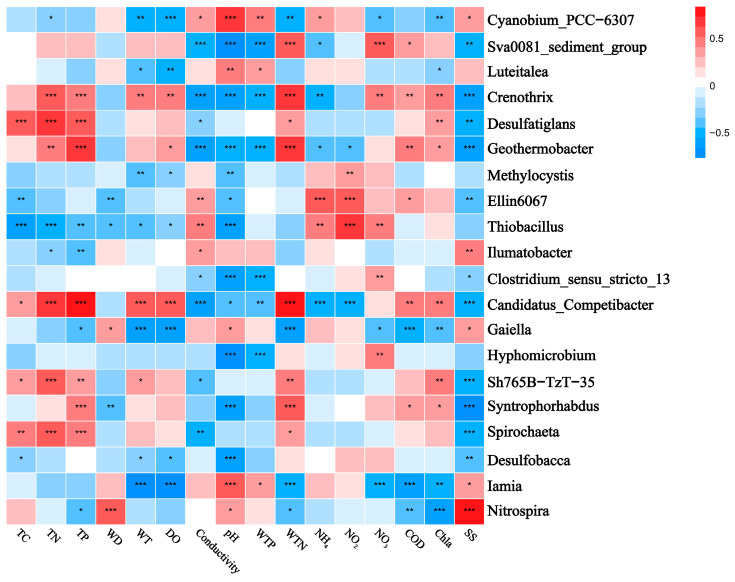
Correlation heatmap showing the relationship between the top-ranked bacterial genera and water and sediment physicochemical properties across different lakes. Positive and negative correlations are indicated in red and blue, respectively (‘*’ indicates 0.01 < *p* ≤ 0.05; ‘**’ indicates 0.001 < *p* ≤ 0.01; ‘***’ indicates *p* ≤ 0.001). The correlations were calculated using the Pearson correlation coefficient. TC: total carbon in sediment; TN: total nitrogen in sediment; TP: total phosphorus in sediment; WT: water temperature; DO: dissolved oxygen; Conductivity: electrical conductivity; pH: pondus hydrogen; WTP: total phosphorus in water; WTN: total nitrogen in water; NH_4_^+^: ammonia nitrogen; NO_3_^−^: nitrate nitrogen; NO_2_^−^: nitrous nitrogen; COD: chemical oxygen demand; Chla: chlorophyll a; SS: suspended solids.

The correlation heatmaps in Figure 7 and Figure 8 illustrate the relationship between the bacterial community and environmental factors, showing the magnitude and significance of correlations between various environmental variables and bacterial genera or phyla. At the phylum level, Pseudomonadota exhibited a significant negative correlation with pH, while Chloroflexota was positively correlated with TP and WTN and negatively correlated with conductivity. Bacteroidota showed a negative correlation with pH and SS, whereas Verrucomicrobia was positively correlated with WTN and COD. Additionally, Planctomycetota displayed a negative correlation with conductivity and a positive correlation with TC and TN. At the genus level, *Luteialea* was negatively correlated with DO and Chla and positively correlated with pH and WTP. *Thiobacillus* showed a negative correlation with conductivity and positive correlations with pH, TC, TN, and TP.

**Figure 9:**
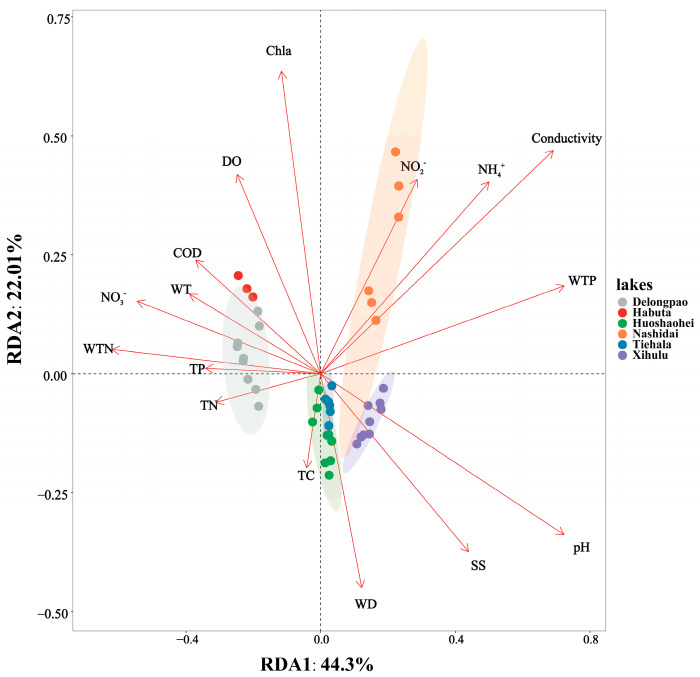
RDA combining correspondence analysis with multiple regression analysis, where each step is regressed against environmental factors. Each dot represents a sample, with different colored dots corresponding to different lakes and ellipses indicating the 95% confidence intervals for each lake. The proximity of two dots reflects the higher functional similarity between the samples. Arrows represent environmental factors, and the angle between two arrows indicates the magnitude of the correlation between them. An acute angle suggests a positive correlation, a right angle suggests no correlation, and an obtuse angle indicates a negative correlation. The length of an arrow indicates the strength of that factor’s influence on the bacterial community structure and function. The angle between the arrow and the coordinate axis represents the degree of correlation with that axis, with lower angles indicating a higher correlation. The projection of a sample onto an environmental factor arrow reflects the relative value of that factor in the sample. The percentages next to the axes represent the proportion of variance in the dataset explained by each axis. TC: total carbon in sediment; TN: total nitrogen in sediment; TP: total phosphorus in sediment; WT: water temperature; DO: dissolved oxygen; Conductivity: electrical conductivity; pH: pondus hydrogen; WTP: total phosphorus in water; WTN: total nitrogen in water; NH_4_^+^: ammonia nitrogen; NO_3_^−^: nitrate nitrogen; NO_2_^−^: nitrous nitrogen; COD: chemical oxygen demand; Chla: chlorophyll a; SS: suspended solids.

RDA revealed the relationships between environmental factors and sediment bacterial communities across different lakes. The two axes accounted for 66.31% of the total variation, with the first RDA axis (RDA1) explaining 44.3% and the second axis (RDA2) accounting for 22.01%. The sediment bacterial communities of Delongpao Lake were positively correlated with TN, TP, WTN, NO_3_^−^, WT, and COD. In contrast, the community in Habuta Lake showed positive correlations with DO, Chla, COD, WT, NO_3_^−^, WTN, TP, and TN. The bacterial communities of Huoshaohei and Tiehala lakes were positively correlated with TC and WD, while Xihulu Lake’s bacterial communities were positively correlated with pH and SS. Lastly, Nashidai Lake’s bacterial communities were positively correlated with NO_2_^−^, NH_4_^+^, conductivity, and WTP (Figure 9).

## 4. Discussion

### 4.1. Variations in the Structure of Sediment Bacterial Communities in Different Lakes

Bacteria in lakes play crucial roles in various biogeochemical cycles, particularly in breaking down organic matter into nutrients for other organisms [46]. They are also key players in nutrient remineralization and pollutant degradation, serving as vital food and nutrient sources for aquatic life, thereby influencing the balance of biological and abiotic systems within lakes. Microbial diversity, which includes genetic, species, and ecosystem-level variations, is often measured by alpha diversity, combining species richness and evenness as indicators of community stability [47].

In our study, significant differences were observed in the physicochemical properties and sediment bacterial communities among the six lakes These differences likely arise from factors such as variations in water flow, human activities, and nutrient inputs [48]. The high levels of DO and Chla in Nashidai Lake suggest a high rate of photosynthetic activity, likely driven by nutrient enrichment. Moreover, the TN and TP contents in several lakes, including Habuta, Xihulu, Nashidai, and Tiehala, exceeded China’s “Class III” water quality standards (1.0 and 0.05 mg/L, respectively). In the Delongpao and Huoshaohei lakes, TN and TP exceeded the “Class IV” standards (1.5 and 0.1 mg/L), suggesting varying degrees of eutrophication in the six lakes of Lianhuan Lake [49]. McCormick et al. [50] demonstrated that nutrient enrichment could substantially affect phytoplankton, leading to increased Chla concentrations. This could explain the elevated Chla levels in Nashidai Lake, likely due to nutrient inputs from agricultural runoff, sewage discharge, and urban runoff, which supply essential nutrients such as nitrogen and phosphorus for phytoplankton growth [51,52].

Additionally, significant differences in bacterial alpha diversity were detected across the six lakes (Figure 2). The Shannon index, which estimates both bacterial richness and evenness, indicates that Tiehala Lake not only hosts a diverse range of species but also maintains a balanced microbial community. The ACE and Chao1 indices, which measure species richness, suggest that Tiehala and Delongpao lakes may harbor more species. On the other hand, the low ACE index in Nashidai Lake suggests fewer species, which may be due to higher nutrient levels (e.g., COD, NH_4_^+^, and WTP) inhibiting microbial growth, thereby reducing overall species richness. Nashidai Lake had the highest Chla levels among the six lakes studied (Table 1), and Chla is widely recognized as an indicator of eutrophication, directly correlating with phytoplankton biomass and nutrient enrichment [53,54,55]. High nutrient concentrations often lead to eutrophication [56], which favors fast-growing species that outcompete others, resulting in reduced species diversity [57].

Additionally, the significant differences in sediment bacterial community structures, as revealed by NMDS analyses (Figure 3) and PERMANOVA analyses (Table 3), point to distinct environmental drivers influencing the microbial communities in each lake. For example, due to their geographic proximity, Delongpao and Habuta lakes are both primarily influenced by TN, TP, WTN, NO_3_^−^, WT, and COD (Figure 9). In contrast, the other four lakes, which are also geographically close to one another, are affected mainly by DO, Chla, COD, WT, NO_3_^−^, WTN, TP, and TN. These variations in environmental conditions likely contribute to the differences in sediment microbial communities between the lakes. Moreover, there was no significant difference in sediment bacterial community structure between the Huoshaohei and Tiehala lakes. This similarity can be attributed to their geographic proximity and physical connection, resulting in closely related bacterial communities with no significant structural changes.

The dominant phyla identified across the lakes, such as Pseudomonadota, Chloroflexota, Actinobacteria, and Acidobacteria (Figure 4), align with findings from previous research on aquatic microbial community structures [58,59,60,61,62,63]. For example, Yi et al. (2021) found that Chloroflexota and Actinobacteria were the dominant phyla in Baiyangdian Lake in northern China [62]. Similarly, Tang et al. (2021) reported that Actinobacteria were dominant in two freshwater lakes in northeastern China [63]. These studies demonstrate that Actinobacteria are a prominent component of lake bacterial communities across various geographical regions and environmental conditions. Previous research has emphasized the role of Chloroflexota in degrading organic pollutants, cycling carbon, and facilitating biogeochemical processes in aquatic ecosystems. Furthermore, we observed a significant abundance of unclassified bacteria in the Lianhuan lakes, which is common in microbial community analyses. To refine our results, we screened out unclassified bacterial genera. Among the classified taxa, Anaerolineaceae, a family within the Chloroflexota phylum, exhibited relatively high abundance across all regions. This family plays a crucial role in anaerobic or microaerobic environments, where it decomposes carbon compounds and contributes to methane production in anaerobic microbial systems.

Bioindication has become crucial for water quality monitoring by utilizing the presence and abundance of bioindicator taxa, primarily multicellular eukaryotes, for biotic indices [64]. However, bacteria are rarely used as bioindicators in routine assessments, despite their recognized importance in environmental processes. Particularly, Cyanobacteria are notable indicators due to their ability to produce toxins and odors that negatively impact water quality and pose significant threats to water resources [65]. In our study, Cyanobacteria were abundant across all six lakes. Notably, the abundance of *Cyanobium PCC-630*, a member of the Cyanobacteria phylum, increased in the downstream lakes (Xihulu, Nashidai, Huoshaohei, and Tiehala) of the Lianhuan Lake system. This suggests that eutrophication is more pronounced in the downstream regions of the Lianhuan lakes.

### 4.2. Relationships between Sediment Bacterial Communities and Physicochemical Properties of Sediment and Water

The observed differences in sediment bacterial communities across lakes could be influenced by variations in sediment and physicochemical properties. The Mantel analyses (Figure 6) indicated that bacterial community diversity and richness are primarily affected by conductivity and chlorophyll a (Chla), which is consistent with several previous studies. Notably, conductivity emerged as the most strongly correlated factor, consistent with prior research. For example, Reid et al. (2021) identified variations in EC as a key driver of biodiversity [66], while Mai et al. (2022) demonstrated a positive correlation between conductivity and bacterial richness [67]. Conductivity is closely linked to salinity, an important environmental factor regulating lake bacterial communities. Previous studies have shown that salinity significantly reduces bacterial abundance [63], diversity, and evenness in lakes [68]. This may be due to salinity not only affecting bacterial growth and abundance but also serving as a physiological barrier for certain bacteria, limiting their survival and reproduction, which leads to decreased species diversity [63].

Chla, a crucial indicator of water eutrophication [69], was also found to influence bacterial communities. Kiersztyn et al. (2019) found that lakes with higher eutrophication levels exhibit higher bacterial diversity, as measured by the Simpson index [70]. This could be attributed to the greater overall bacterial productivity in eutrophic lakes, driven by higher primary productivity, which increases bacterial richness. However, excessive eutrophication can lead to biodiversity loss [71].

Additionally, Mantel analyses highlighted significant correlations between sediment bacterial community composition and factors such as sediment TP, pH, and WTN [22,72], corroborating findings from previous studies [73,74]. Nitrogen and phosphorus are critical for microbial communities, influencing cell abundance and activity. The concentration of these nutrients affects nutrient cycling, alters bacterial growth rates, and subsequently shifts community composition [75,76]. Water pH also plays a vital role by controlling the solubility of metal ions in aquatic ecosystems, with previous studies showing that bacterial community composition varies with metal ion concentrations in water bodies [77,78].

The heatmaps further demonstrate the correlations between environmental factors and sediment bacterial communities. At the phylum level, the results showed that conductivity positively correlates with Actinobacteria and negatively correlates with Verrucomicrobiota, while Cyanobacteria positively correlate with pH and Chloroflexota correlate with WTN. These findings are consistent with previous studies [79,80,81]. For instance, Meng et al. (2020) found a positive correlation between Actinobacteria and electrical conductivity in groundwater [81], while Dao et al. (2016) reported a positive correlation between Cyanobacteria biomass and pH levels [82]. This might be due to certain Cyanobacteria preferring HCO_3_^−^ over CO_2_ as a carbon source [83].

## 5. Conclusions

Our research in Lianhuan Lake, located in Northeast China, has shed light on how different lake environments impact sediment bacterial communities. As expected, we observed significant changes in both the alpha and beta diversity of these communities, influenced by varying lake conditions. These changes were accompanied by differences in the relative abundance of specific bacterial genera. For example, the relative abundance of the dominant bacterial phyla varied between lakes, with Pseudomonadota being most abundant in Habuta Lake and least abundant in Xihulu Lake, while Chloroflexota was most abundant in Tiehala Lake and least abundant in Habuta Lake. The environmental factors influencing each lake’s sediment bacterial community structures were distinct. For instance, the bacterial communities in Delongpao and Habuta Lakes were correlated with TP, TN, WT, COD, NO_3_^−^, and WTN, while those in the other four lakes were associated with conductivity, WTP, pH, SS, WD, NH_4_^+^, and NO_2_^−^. This study constitutes the first comprehensive assessment of the variations in bacterial communities in different lakes across the entire Lianhuan Lake system. Our findings provide important insights into the stability of lake ecosystems and offer valuable contributions to microbial ecology. They also serve as a critical resource for the management and conservation of freshwater ecosystems.

## Figures and Tables

**Table 1:** Physical and chemical variables of the water samples from the six sites of Lianhuan Lake.

The Name of the Lake	WT (°C)	DO (mg L^−1^)	pH	Conductivity (μs cm^−1^)	COD (mg L^−1^)	NH_4_^+^-N (mg L^−1^)	WTP (mg L^−1^)	WTN (mg L^−1^)	NO2–N (mg L^−1^)	NO3–N (mg L^−1^)	Chla (μg L^−1^)	SS (mg L^−1^)
Habuta	22 ± 0.5 b	7.59 ± 0.02 c	7.84 ± 0.01 c	654 ± 1 b	10 ± 1 a	1.5 ± 0.01 a	0.1 ± 0.01 c	1.48 ± 0.01 b	0.14 ± 0 a	0.17 ± 0 a	16.3 ± 0.01 ab	11 ± 0 b
Delongpao	24.03 ± 0.95 a	9.93 ± 0.15 a	8.44 ± 0.17 b	531.5 ± 50.1 d	13.36 ± 1.71 a	1.01 ± 0.17 c	0.12 ± 0.01 c	2.16 ± 0.34 a	0.07 ± 0.02 c	0.11 ± 0.01 b	21.08 ± 4.18 ab	10.67 ± 3.21 b
Xihulu	21.5 ± 0.1 b	7.36 ± 0.33 c	8.89 ± 0.04 a	657.33 ± 6.03 b	5.85 ± 0.37 a	1.43 ± 0.16 a	0.16 ± 0.03 bc	1.37 ± 0.28 b	0.1 ± 0.01 b	0.06 ± 0.01 c	15.34 ± 1.61 ab	16.67 ± 3.06 ab
Nashidai	23.4 a	9.95 ± 0.92 a	8.79 ± 0.2 a	755 ± 1.41 a	12.14 ± 6.77 a	1.64 ± 0.13 a	0.18 ± 0.01 a	1.45 ± 0.05 b	0.12 ± 0.01 ab	0.1 ± 0.02 bc	21.85 ± 5.63 a	17.5 ± 0.71 ab
Huoshaohei	23.07 ± 0.15 a	9.01 ± 0.06 b	8.71 ± 0.02 a	596.33 ± 0.58 c	6.97 ± 1.31 a	1.03 ± 0.19 bc	0.13 ± 0.01 c	1.5 ± 0.16 b	0.08 ± 0.01 c	0.1 ± 0.03 bc	13.16 ± 3.39 c	24.67 ± 6.51 a
Tiehala	23.3 ± 0.14 a	8.73 ± 0.18 b	8.67 ± 0.01 ab	595 ± 1.41 c	11.38 ± 7.84 a	1.36 ± 0.07 ab	0.13 ± 0.01 bc	1.43 ± 0.08 b	0.09 ± 0.01 bc	0.09 ± 0 bc	16.56 ± 3.18 ab	17.5 ± 4.95 ab

Values are reported as mean ± standard error; different letters represent significant differences between treatments (*p* < 0.05). WT: water temperature; DO: dissolved oxygen; Conductivity: electrical conductivity; pH: *pondus hydrogenii*; WTP: total phosphorus in water; WTN: total nitrogen in water; NH_4_^+^-N: ammonia nitrogen; NO_3_^−^-N: nitrate nitrogen. NO_2_^−^-N: nitrous nitrogen; COD: chemical oxygen demand; Chla: chlorophyll a; SS: suspended solids. Significant variations between experimental groups were identified via ANOVA, followed by the Waller–Duncan post hoc test for pairwise comparisons.

**Table 2:** Chemical variables of the sediments from the six sites examined within the Lianhuan Lake watershed.

The Name of the Lake	TC (g/kg)	TN (g/kg)	TP (g/kg)
Habuta	6.65 ± 0.21 b	0.61 ± 0.02 c	0.28 ± 0.04 c
Delongpao	39.15 ± 13.64 a	3.55 ± 1.23 a	1.46 ± 0.61 a
Xihulu	28.80 ± 6.78 a	1.89 ± 0.37 b	0.78 ± 0.17 b
Nashidai	28.13 ± 1.89 a	1.96 ± 0.15 b	0.76 ± 0.13 b
Huoshaohei	34.59 ± 11.16 a	2.28 ± 0.57 b	0.66 ± 0.06 bc
Tiehala	29.64 ± 0.87 a	2.13 ± 0.09 b	0.76 ± 0.15 b

Values are reported as mean ± standard error; different letters within each column indicate significant differences between treatments (*p* < 0.05). TC: total carbon; TN: total nitrogen; TP: total phosphorus. Significant variations between experimental groups were identified using ANOVA, followed by the Waller–Duncan post hoc test for pairwise comparisons.

**Table 3:** PERMANOVA analysis of bacterial community composition across different lakes.

Pairs	R^2^	*p*-Value	*p*-Adjusted
Habuta vs. Delongpao	0.6395955	0.01	0.01154
Habuta vs. Xihulu	0.6867442	0.01	0.01154
Habuta vs. Nashidai	0.6458678	0.018	0.01929
Habuta vs. Huoshaohei	0.5856005	0.005	0.00750
Habuta vs. Tiehala	0.6683037	0.009	0.01154
Delongpao vs. Xihulu	0.7043276	0.001	0.00188
Delongpao vs. Nashidai	0.6667118	0.001	0.00188
Delongpao vs. Huoshaohei	0.5943315	0.001	0.00188
Delongpao vs. Tiehala	0.5958403	0.001	0.00188
Xihulu vs. Nashidai	0.4028978	0.001	0.00188
Xihulu vs. Huoshaohei	0.3039818	0.001	0.00188
Xihulu vs. Tiehala	0.3574453	0.001	0.00188
Nashidai vs. Huoshaohei	0.4123211	0.001	0.00188
Nashidai vs. Tiehala	0.4333769	0.002	0.00333
Huoshaohei vs. Tiehala	0.1173916	0.063	0.06300

## Data Availability

All data pertaining to this study are contained within the article.

## Supplementary Materials

The following supporting information can be downloaded at https://www.mdpi.com/article/10.3390/microorganisms12091914/s1, Figure S1: The Sampling Procedure for Sediment Bacterial Communities in Six Lakes.
