# Supplementary figures and images for: Bacterial Diversity in Sediments from Lianhuan Lake, Northeast China
Source: Microorganisms. 2024 Sep 20;12(9):1914. doi: 10.3390/microorganisms12091914 (PMC11433699; doi:10.3390/microorganisms12091914)

Figure S1. The Sampling Procedure for Sediment Bacterial Communities in Six Lakes

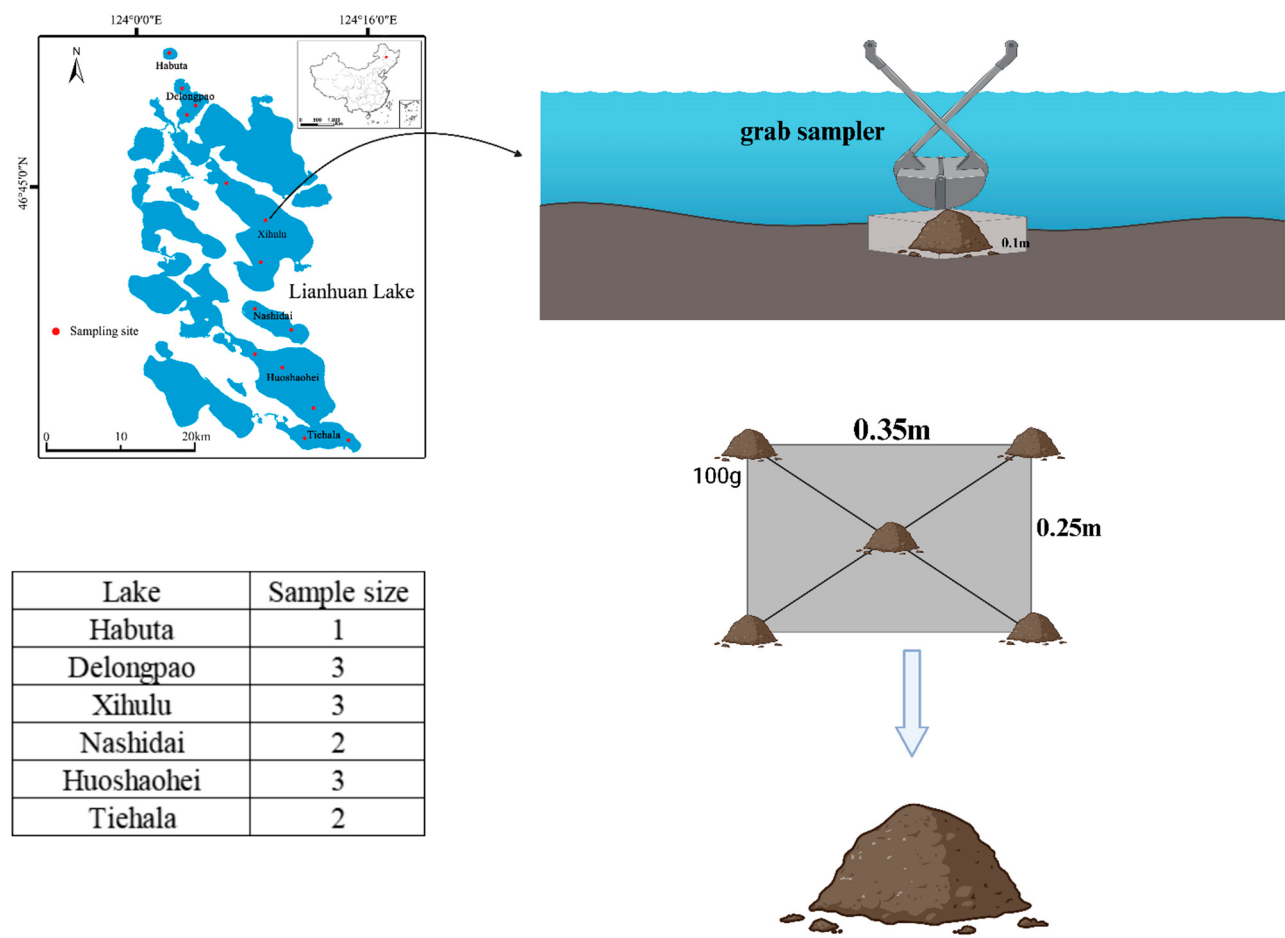

Supplement: Supplementary file 1 [file microorganisms-12-01914-s001.zip › microorganisms-3191073-supplementary.pdf]
